# A synthetic peptide from Sipunculus nudus promotes bone formation via Estrogen/MAPK signal pathway based on network pharmacology

**DOI:** 10.3389/fphar.2023.1173110

**Published:** 2023-04-24

**Authors:** Peiran Wang, Zhenhui Feng, Siyu Chen, Yingye Liang, Haiyan Hou, Qianqian Ouyang, Hui Yu, Hua Ye, Lei Cai, Yi Qi, Kefeng Wu, Hui Luo

**Affiliations:** ^1^ Marine Biomedical Research Institution, Guangdong Medical University, Zhanjiang, China; ^2^ Guangdong (Zhanjiang) Provincial Laboratory of Southern Marine Science and Engineering, Zhanjiang, China; ^3^ Marine Traditional Chinese Medicine Sub-center of National Engineering Research Center for Modernization of Traditional Chinese Medicine, Zhanjiang, China; ^4^ Guangdong Key Laboratory for Research and Development of Natural Drugs, Guangdong Medical University, Zhanjiang, China; ^5^ Guangdong Provincial Key Laboratory of Laboratory Animals, Guangdong Laboratory Animals Monitoring Institute, Guangzhou, China

**Keywords:** sipunculus nudus, zebrafish, bone development, network pharmacology, estrogen signaling pathway, MAPK signaling pathway

## Abstract

The tripeptide Leu-Pro-Lys (LPK), derived from the Sipunculus nudus protein, was synthesized and studied to investigate its potential protective effect on bone formation. The effect and mechanism of LPK were analyzed through network pharmacology, bioinformatics, and experimental pharmacology. The study found that LPK at concentrations of 25 μg/mL and 50 μg/mL significantly increased ALP activity and mineralization in C3H10 cells. LPK also increased the expression of COL1A1 and promoted bone formation in zebrafish larvae. Network pharmacology predicted 148 interaction targets between LPK and bone development, and analysis of the protein-protein interaction network identified 13 hub genes, including ESR1, MAPK8, and EGFR, involved in bone development. Through KEGG enrichment pathways analysis, it was determined that LPK promotes bone development by regulating endocrine resistance, the relaxin signaling pathway, and the estrogen signaling pathway. Molecular docking results showed direct interactions between LPK and ESR1, MAPK8, and MAPK14. Additional verification experiments using western blot assay revealed that LPK significantly upregulated the expression of genes related to bone formation, including COL1A1, OPG, RUNX2, ESR1, phosphorylated MAPK14, and phosphorylated MAPK8 in C3H10 cells. These results suggest that LPK promotes bone formation by activating the estrogen/MAPK signaling pathway.

## 1 Introduction

Sipunculus nudus is a commonly consumed seafood in the north bay area of China that is known to regulate gastrointestinal function and promote sperm motility (Zhang et al., 2011). This is consistent with traditional Chinese medicine’s theory of “tonifying the kidney and consolidating essence” (Wang et al., 2012). Traditional Chinese medicine also posits that the “kidney governs the bone,” suggesting that compounds derived from Sipunculus nudus may benefit osteoporosis and bone formation. The high crude protein content (over 70%) (Dong et al., 2012) of Sipunculus nudus suggests that the anti-osteoporotic effects may be related to its protein and peptide fractions.

Small peptides obtained from enzymatic digestion have become a popular area of research due to their higher absorption level (Zheng, 2010). They have better drug-forming properties as compared to larger peptides as the fewer peptide bonds on small peptides make them difficult for gastrointestinal proteases to recognize and are more resistant to degradation by digestive enzymes (Zhang, 2003).

Our preliminary study (Liu et al., 2021) revealed that LPK, an active tripeptide obtained by enzymatic cleavage of Sipunculus nudus proteins, has strong antioxidant properties and is involved in the synthesis of several proteins such as R7UNS2, R7UBD8, R7UTA6, R7TB03, R7V7P3, R7V3Y8, A3QRJ2, etc., which play critical roles in cell membrane composition, enzyme activation, binding to essential biomolecules, and other cell growth and differentiation activities. For example, *in vivo* studies revealed that the R7TB03 protein is involved in the positive regulation of the IKK/NFKB signaling pathway (Jeon et al., 2021) and in the activation of MyD88-dependent Toll-like receptor signaling pathways, all of which are involved in promoting osteoblast differentiation. The R7V7P3 protein is reportedly related to G protein-coupled receptor activity. Studies have shown that when the G protein-coupled receptor is inhibited, the proliferation of osteoblasts is reduced (Wu et al., 2010). Previous studies have also demonstrated that the combination of estrogen and the G protein-coupled receptors can trigger MAPK signal transduction and ERK cascade activation (Kousteni et al., 2001), thereby promoting cell growth and proliferation. The above results suggest that LPK has good potential for promoting bone formation.

In this study, we aim to investigate the effects of Sipunculus nudus tripeptide (LPK) on bone formation. We established an osteogenic induction cell model using mouse mesenchymal stem cells and used zebrafish larvae as laboratory animals for *in vivo* tests. We used network pharmacology to identify the essential targets and pathways of LPK in promoting osteogenesis, performed molecular docking simulation evaluations, and conducted western blotting experiments to explore and clarify the potential mechanism.

## 2 Materials and methods

### 2.1 Chemicals and reagents

Calcein and tricaine methanesulfonate were purchased from Sigma-Aldrich (St. Louis, MO, United States). Polyoxymethylene (POM) was purchased from Beijing Leagene Biotech Co., Ltd. (Beijing, China). 75% ethyl alcohol and 3% hydrogen peroxide disinfectant were provided by Shandong LIRCON Medical Technology Incorporated Company (Shandong, China). 95% ethyl alcohol, absolute alcohol, and Ethylenediaminetetraacetic acid (EDTA) were purchased from Guangdong Guanghua Sci-Tech Co., Ltd. (Guangdong, China). Phosphate buffered saline (PBS)was purchased from Hyclone Laboratories Inc. (Logan, UT, United States). Imidazole and ascorbic acid were purchased from Macklin Inc. (Shanghai, China). 5% BSA Blocking Buffer, 0.2% Alizarin Red S Staining Solution, and 0.25% Trypsin-EDTA solution were provided by Beijing Solarbio Science & Technology Co., Ltd. (Beijing, China). Dulbecco’s Modified Eagle Medium (DMEM), fetal bovine serum (FBS), and penicillin-streptomycin were purchased from Life Technologies Life Sciences Solutions Group, Thermo Fisher Scientific (Waltham, MS, United States). β-Glycerophosphate disodium salt hydrate (BGP) was purchased from Hefei Bomei Biotechnology Co., Ltd. (Anhui, China). Dexamethasone Sodium Phosphate Injection was purchased from Shanxi Ruicheng Kelong Veterinary Medicine Co., Ltd. (Shanxi, China). BCIP/NBT Alkaline Phosphatase Color Development Kit was purchased from Beyotime Biotech. Inc. (Shanghai, China). Mouse monoclonal ZNS-5 to Osteoblast Marker was purchased from Abcam (Cambridge, United Kingdom). COL1A1 Antibody was purchased from Cell Signaling Technology (Danvers, MS, United States). DyLight™ 405 AffiniPure Goat Anti-Rabbit IgG (H + L) and Alexa Fluor^®^ 680 AffiniPure Goat Anti-Rabbit IgG were provided by Jackson Immuno Research (Sigrove, PA, United States).

### 2.2 Preparation, structure determination, and Lipinski’s Rule of Five determination of sipunculus nudus synthetic peptide (LPK)

The synthetic peptide Sipunculus nudus (LPK) was produced by DGpeptides Co., Ltd. (Zhejiang, China) according to the amino acid sequence (-Leu-Pro-Lys-). The high-performance liquid chromatography (HPLC) conditions used were as follows: a Boston Green ODS-AQ Column (250 mm × 4.6 mm, Boston Analytics, United States) was used with mobile phase A consisting of 0.1% formic acid and 2% acetonitrile, and mobile phase B consisting of 0.1% formic acid and 100% acetonitrile. The flow rate used was 1.0 mL/min and elution gradients were as follow: 0–8 min, 6% B; 8–24 min, 9% B; 24–60, 14% B; 60–75 min, 30% B; 75–78 min, 40% B; 78 min, 95% B.

The mass spectrometer was used under the following conditions: MS resolution: 70,000@m/z; AGC: 3e6; MaximumIT: 40 ms; MS precursor m/z range: 300.0–1,600.0. MS/MS parameters: resolution: 17,500@m/z; AGC: 1e5; MaximumIT: 60 ms; TopN: 20; NCE/steppedNCE: 27.

Lipinski’s Rule of 5 (Ro5) is a criterion used to assess the likelihood of a biologically active molecule to have the chemical and physical properties required for oral bioavailability. It includes five assessment factors: molecular weight (MW), octanol-water partition coefficient (AlogP), polar surface area (TPSA), hydrogen bond donors (Hdon), and hydrogen bond acceptors (Hacc). The drug-likeness of LPK was evaluated using Lipinski’s Rule of Five (http://www.scfbio-iitd.res.in/software/drugdesign/lipinski.jsp) (Lipinski, 2004).

### 2.3 Prediction of potential targets of synthetic peptide (LPK) of sipunculus nudus

Open Babel (O'Boyle et al., 2011) was used to convert the molecular formula of LPK into a SMILES string, which was then imported into UCSF Chimera software (v.1.16) (Pettersen et al., 2004) to generate the most stable 3D structure. Potential targets for LPK were predicted using PharmMapper, (http://www.lilab-ecust.cn/pharmmapper/) (Wang et al., 2017), and hit target pharmacophore models were listed by normalized fit score, discarding those with scores less than 0.5. Using the UniProt database (https://www.UniProt.org/) (Consortium, 2019), human genes from the identified targets were identified.

### 2.4 Screening of related targets for bone-promoting effects

Related targets for bone-promoting effects were retrieved from the Genecards (https://www.genecards.org/) (Stelzer et al., 2011) and DisGeNET (http://www.disgenet.org/web/DisGeNET/menu/home) (Piñero et al., 2019) databases by using keywords such as “bone formation,” “bone density,” and “osteoporosis” to screen targets. Targets correlated with bone-promoting were obtained by eliminating repetitive targets. The overlapping targets were obtained using the Jvenn website (http://jvenn.toulouse.inra.fr/app/index.html) (Bennett et al., 2016), and the ‘target-target’ (T-T) network, which relates LPK and bone-promoting effects, was constructed using Cytoscape software (v.3.9.1) (Shannon et al., 2003).

### 2.5 Protein-protein interaction (PPI) network and hub gene

To deeply explore the mechanism of LPK in a bone-promoting way, the STRING database (https://cn.string-db.org/) (Szklarczyk et al., 2021) was used to identify protein interactions related to LPK. And a Protein-Protein Interaction (PPI) network was created using Cytoscape software. A high confidence score of 0.70 was used to filter out less qualified genes.

Three Cytoscape plug-ins were utilized to identify hub genes from the PPI network. The degree and betweenness (Bonte et al., 2021) of each node within the network were calculated using CentiScaPe 2.2 (Scardoni et al., 2014) and those genes with values greater than the mean were collected as potential hub genes. The relationship between nodes and sides were analyzed using Mcode (Bader and Hogue, 2003) and the subnetwork with the highest score was considered as a potential hub gene source. The top 20 most important nodes in the PPI network based on Maximal Clique Centrality scores (MCC) were identified using CytoHubba (Chin et al., 2014). Finally, an intersection of the results from all three methods was taken to obtain a final list of hub genes.

### 2.6 Biological pathway enrichment analysis

The final list of hub genes was uploaded to the Metascape platform (https://metascape.org/) (Zhou et al., 2019) to analyze the mechanism of LPK in relation to bone promotion. KEGG enrichment pathway analysis (Kanehisa et al., 2022) was performed to investigate the biological functions of the hub genes and the top 20 pathway terms were selected for functional annotation. The results were visualized using the ggplot2 (Wickham, 2016) plug-in in R Studio. Additionally, ClueGO (Bindea et al., 2009), a Cytoscape plug-in, was used to visualize the biological functions of the hub genes, with different colors used to distinguish the results from different hub gene sources.

### 2.7 Molecular docking

To understand how LPK binds to protein targets to produce therapeutic effects, molecular docking was performed using the Glide module of the Schrödinger suite (Friesner et al., 2006). Based on the results from the PPI network and KEGG enrichment pathways, ESR1, MAPK14, and MAPK8 were selected as candidate protein targets for LPK. The 3D structure of the LPK was generated using Open Babel and energy-minimized with UCSF Chimera software (v1.16). The 3D structures of ESR1 (PDB ID: 7B9R) (Sijbesma et al., 2021), MAPK14 (PDB ID: 2FST) (Diskin et al., 2007), and MAPK8 (PDB ID: 2XRW) (Garai et al., 2012) were downloaded from the PDB database (https://www.rcsb.org/) (Berman et al., 2000).

Since the protein structure files obtained from the PDB database were not suitable for molecular docking, the Protein Preparation Wizard tool (Madhavi Sastry et al., 2013) was used to prepare suitable structures. The tool was used to remove ligand water molecules, add hydrogen atoms, assign protonation states and partial charges, refine target structures, build the molecular environment force fields, and use OPLS 2005 as an impromptu solvent for rigid protein energies. The final protein-peptide complex energy was minimized from the interaction energy (Eint). The scoring function for Glide Docking was computed from the total energy model (E-model) ranking of each ligand. The E-model is the sum of the Glide Score, non-bonded interaction energies, and the excess internal energy of the complex.

### 2.8 Animals and experimental grouping

In this study, adult wild-type AB-strain zebrafish were used as the model organisms. The AB-strain zebrafish were obtained from the Type AB Zebrafish Platform Center of the Guangdong Medical University Affiliated Hospital. The study was conducted in accordance with the procedures outlined in the *Type AB Zebrafish Handbook* and in accordance with the *Guide for the Care and Use of Laboratory Animals*.

The male and female zebrafish were randomly divided into groups for natural mating. Fertilized eggs produced were collected, placed in cell-culture dishes, and then incubated in a solution of egg water (5 mmol/L NaCl, 0.17 mmol/L KCl, 0.4 mmol/L CaCl2, 0.16 mmol/L MgSO4, and 10ppm methylene blue) in a thermostat incubator at a temperature of 28.5 ± 0.5°C, pH 7–7.2. The eggs were kept under a 14 h:10 h day/night cycle.

The LPK from Sipunculus nudus in powder form was dissolved in egg water to prepare solutions with LPK concentrations of 12.5, 25, and 50 μg/mL. These solutions were used to treat zebrafish larvae in three groups with different concentrations.

Three days post-fertilization (3 dpf), the zebrafish larvae were transferred to four wells of a six-well plate (2 × 3), each containing 4 mL of egg water. 20 randomly selected larvae were placed in each well and divided into a blank/control group and experimental groups (12.5, 25, and 50 μg/mL). The egg water in each well was then replaced with the prepared LPK solutions. The solution in each well was replaced with an equal volume of new solution every day and the plate was maintained in a thermostat incubator at 28.5 ± 0.5°C. The culture cycle was 6 days. The zebrafish larvae were not fed during the culture process. At 9 dpf, 15 fish with similar characteristics were selected from the four fish groups for testing.

### 2.9 Calcein assay

Calcein is a dye that is commonly used for *in vivo* staining of living cells or small animals, like zebrafish. It binds to calcium ions in tissues and emits green fluorescence when viewed under a confocal microscope. This fluorescence is used to indicate the degree of bone development and mineralization in the tissue.

At 9 dpf, the zebrafish larvae were live-stained for about 15 min using calcein dissolved in egg water at a concentration of 0.2%. They were then removed from the staining solution and placed in clean egg water to wash away the excess dye in order to minimize background fluorescence under the microscope.

The larvae were euthanized by placing them in a solution of tricaine dissolved in egg water at a concentration of 0.02%. They were then imaged under a confocal microscope at an excitation wavelength of 488 nm. All images were captured using the same light intensity and exposure settings to ensure consistency.

### 2.10 Alizarin red S (ARS) assay

The staining principle of ARS is similar to that of calcein. A fixed specimen staining approach was selected. The 9 dpf larvae were fixed with 4% paraformaldehyde solution for 6 h. They were then dehydrated using increasing alcohol concentrations (50%, 75%, 95%) for 5 minutes each. The larvae were then placed in a solution of Alizarin Red S overnight. After staining, the larvae were washed twice with PBS. To remove over-stained parts of the tissue, a mixed solution of 1.5% H2O2 and 1% KOH was used. Imaging was performed under the confocal microscope with a 605 nm excitation wavelength using the same settings as before.

### 2.11 Immunofluorescence double assay

The larvae were fixed with 4% paraformaldehyde overnight, and then washed three times with PBS. They were then decalcified for 2 days using a decalcification mixture consisting of 10% EDTA and 15% imidazole. The samples were then dehydrated using a methanol gradient (40%, 60%, 80%, 100%) for 2 hours for each gradient. They were then immersed in a decolorizing agent (a mixture of 30% H2O2 and 100% methanol) overnight. Then rehydrated using an inverse methanol gradient (concentration of methanol opposite to the gradient used for dehydration) for the same amount of time as the dehydration. 2% Triton x100 was used as the infiltration solution for 1 day, then replaced with 5% BSA the next day and left to saturate for 6 h. Finally, the samples were incubated with a double antibody according to a protocol provided by Camila.

### 2.12 Cell culture

The mouse mesenchymal stem cell line (C3H10) was obtained from iCell Bioscience Inc. (Shanghai, China), and was maintained in Dulbecco’s Modified Eagle Medium (DMEM). To induce osteoblast differentiation, C3H10 cells were seeded into 24-well plates (4 × 6) and cultured with prepared osteogenic induction medium (DMEM containing 10% fetal bovine serum, 10 mmol/L β-glycerophosphate, 50 mg/L vitamin C, 1 × 10⁸ mol/L dexamethasone and 1 mL antibiotics) until the cells reached 70% confluency. Then, the osteogenic induction medium containing different concentrations of LPK (12.5, 25, and 50 μg/mL) was added to the culture medium for experimental modeling. Each group had six replicated wells. The medium was replaced every 2 days.

### 2.13 Alkaline phosphatase (ALP) and ARS assays

The cells treated with different concentrations of LPK for about 7 days were separated from the mixture and were fixed with 4% paraformaldehyde solution for 40 min. ALP staining was performed overnight according to the manufacturer’s protocol. The stained cells were then washed twice with PBS, and 0.1% ARS staining was performed using the same procedure as the ALP staining.

### 2.14 Western blot assays

The C3H10 cells were inoculated into 6-well plates and cell modeling was performed as described in 2.12. The cells were cultured until day 7, and the total cellular protein was extracted using RIPA buffer (string) containing a proteinase inhibitor. The cells were lysed in lysis buffer on ice for 1 hour with shaking. The cell debris was precipitated and discarded, and the supernatant was recovered as cell lysate. The cell lysates were used to measure the total protein content using the BCA method. The total protein was mixed with loading buffer to a final concentration of 1× and denatured by heating to 95°C for 15 min.

Protein separation was then performed on a 10% polyacrylamide gel at 100 V for 1.5 h. After separation, proteins were transferred from the gels to PDVF membranes at 0.3 mA for 15 min using trans-blotted SD semi-dry transfer cells. The PDVF membranes were then incubated in 5% skim milk powder for 2 hours at room temperature on an oscillator to block non-specific binding. The PVDF membranes were then incubated with anti-GAPDH, anti-RUNX2, anti-ESR1, anti-MAPK8 (p-MAPK8), anti-MAPK14 (p-MAPK14), anti-Col1α1 and anti-OPG overnight at 4°C. A polyclonal goat-free anti-rabbit IgG-HRP was used as a secondary antibody and incubated with the membrane for 2 hours at 37°C. The strips were exposed by using the chemiluminescence system (Bio–Rad, United States of America).

### 2.15 Statistical analysis

The images obtained were observed by a Spinning Disk Confocal Microscope (OLYMPUS, IXplore-SpinSR), manually quantified, and calculated using Image J software (v.2.1.0). Adobe Photoshop 2020 was used to process and organize the experimental images. The data were analyzed using GraphPad Prism (v.9.0.1) for one-way ANOVA. Adobe Illustrator 2020 was used to produce flowcharts and mechanical diagrams.

## 3 Result

### 3.1 The structural identification of LPK

**FIGURE 1:**
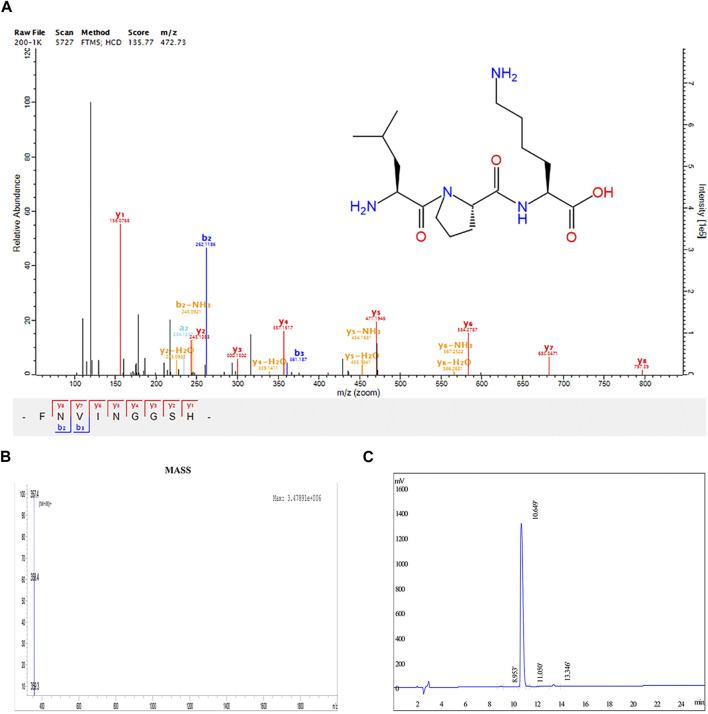
Structural determination results of LPK. **(A)** Chemical structure of LPK. **(B)** HPLC determination results of LPK. **(C)** Mass spectrometry results of LPK.

LPK is a tripeptide compound composed of the amino acids Leucine, Proline, and Lysine (-Leu-Pro-Lys-). It has a relative molecular mass of 356.46 g/mol. Its chemical properties include 4 hydrogen bond donors, 6 hydrogen bond acceptors, 12 rotatable bonds, and a lipid-water partition coefficient of 1.97 (Figure 1A).

The results of HPLC detection showed that LPK has a high resolution and minimal impurity peaks. The sample peak was at 10.649 min (Figures 1B, C).

### 3.2 LPK promotes osteogenic induction and mineralization of cells

**FIGURE 2:**
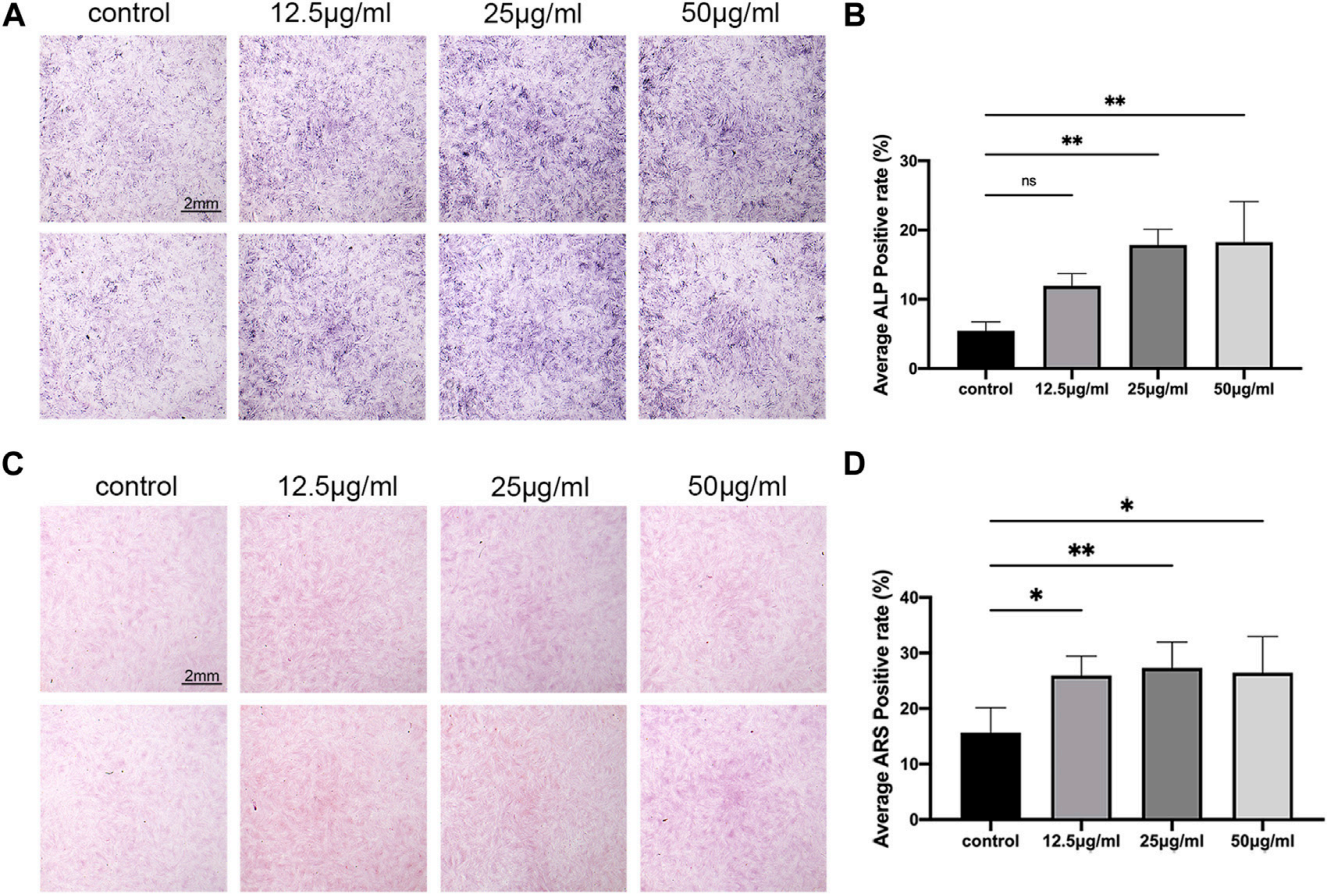
The effect of LPK on osteogenic-induced C3H10 cells *in-vitro*. **(A)** ALP staining of C3H10 cells treated with different concentrations of LPK. **(B)** Quantitative analysis of ALP positive area. The positive rate of ALP was expressed by the detection value under the same condition. Compared to the control, a significant difference was defined at *p* < 0.05 (*); *p* < 0.005 (**); *p* < 0.0005 (***); ns: the difference was not statistically significant. **(C)** ARS staining of C3H10 cells treated with different concentrations of LPK. **(D)** Quantitative analysis of the positive area, compared to the control; the positive rates were considered significantly different at *p* < 0.05 (*); *p* < 0.005 (**).

ALP is one of the marker enzymes of osteoblasts (Li et al., 2017). By staining the 7-day cultured cells with ALP, the differentiation of osteoblasts can be observed. The results of the osteoblast differentiation assay using C3H10 cells revealed that LPK at concentrations of 25 μg/mL and 50 μg/mL significantly increased the positive rate of ALP expression (*p* < 0.005) compared to the control group (Figures 2A, B). Furthermore, the results of ARS staining indicated a significant increase in cell mineralization in all three experimental groups treated with varying concentrations of LPK compared to the control group (*p* < 0.05). Notably, the increase in mineralization was more prominent in the group treated with 25 μg/mL of LPK. (Figures 2C, D). These results indicate that LPK promotes osteoblast differentiation and mineralization in C3H10 cells.

### 3.3 LPK promotes skull mineralization and type I collagen formation in juvenile zebrafish

**FIGURE 3:**
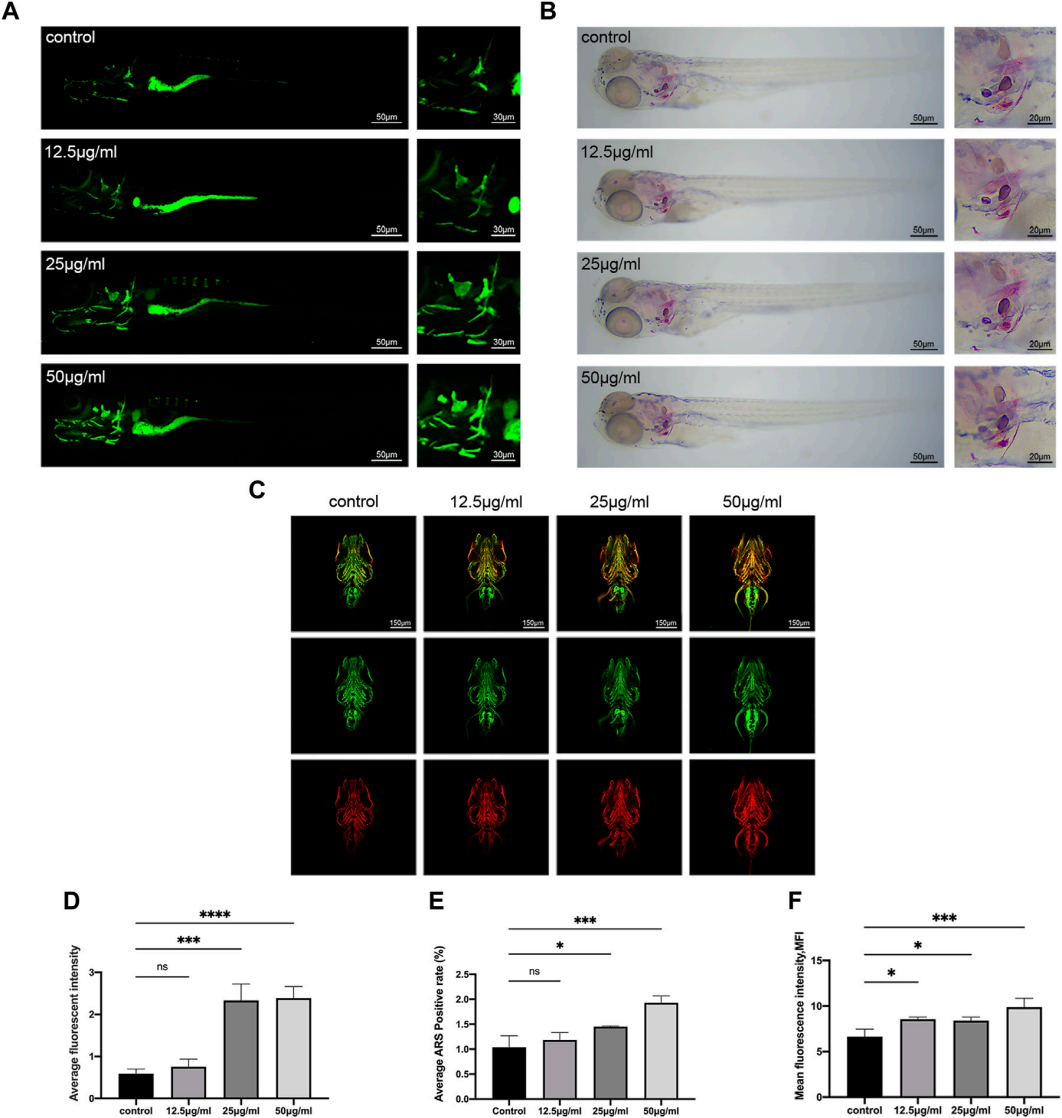
The effect of LPK on zebrafish larvae mineralization *in vivo*. **(A)** *In vivo* calcein staining of 9 dpf zebrafish larvae exposed to different concentrations of LPK under ×4 and ×10 magnification. **(B)** ×4 and ×10 magnified zebrafish larvae with ARS fixed staining. **(C)** Immunofluorescence double staining of zebrafish larvae at ×10 magnification, ZNS5 expressed in green fluorescence and COL1A1 expressed in red fluorescence. **(D)** In average optical density analysis, compared to the control group, optical densities were significantly different at *p* < 0.0005 (***); *p* < 0.0001 (****); ns: the difference was not statistically significant. **(E)** ARS positive area quantitative analysis; compared to the control group, significant differences were defined at *p* < 0.05 (*); *p* < 0.0005 (***); ns: the difference was not statistically significant. **(F)** The average optical density analysis of red fluorescence; compared to the control group, differences were significantly different at *p* < 0.05 (*); *p* < 0.0005 (***).

After the stain of the 9 dpf zebrafish larvae, the results of the staining with calcein and ARS demonstrate that the LPK has the ability to promote the mineralization of the zebrafish skeleton (Figures 3A, B). Comparing the control group to the experimental groups treated with 25 μg/mL and 50 μg/mL, the fluorescence intensity of the calcein staining and the coloration of the ARS staining were found to be significantly deeper (*p* < 0.005) (Figures 3D, E). These findings support the potential of LPK as a potential therapeutic agent for promoting bone formation.

The ZNS5 antibody can be used as a marker of osteoblast differentiation (Wilson and Matsudaira, 1998). After being labeled with a secondary antibody, it exhibits green fluorescence under 488 nm laser excitation. The COL1A1 antibody binds to type I collagen expressed in tissues and is marked with a secondary antibody (Murshed, 2018), which then emits red fluorescence when excited by a 561 nm laser light. The distribution and intensity differences of ZNS5 and COL1A1 expression between each group were compared by measuring the mean fluorescence intensity (MFI) of each group of the two channels, respectively. As shown in Figures 3C, F, compared to the control, the experimental groups treated with 12.5 μg/mL, 25 μg/mL and 50 μg/mL expressed more vigorous fluorescence intensity in the skull part of the larvae of the 561 channel (*p* < 0.005) (Figure 3F), indicating that LPK could promote the expression of COL1A1 in the skull of zebrafish larvae.

### 3.4 Prediction of screening targets and hub genes pathway by network pharmacology

The 3D structure of LPK was analyzed using Pharmmapper, resulting in the identification of 299 potential target proteins. These targets were ranked according to their normalized fit score, with the top 159 scoring at least 0.5 being selected for further analysis. The top ten are presented in Table 1. A search of the Genecards and DisGeNET databases using keywords related to bone density, bone formation, and osteoporosis yielded 12,503, 13,685, and 4,317 protein genes, respectively. Duplicate targets were removed.

**TABLE 1:** Top ten pharmacophore candidates identified by PharmMapper.

Pharma model	Norm fit	Sample	Name	Uniplot
1fh0_v	0.9916	CTSV	Cathepsin L2	CATL2_HUMAN
7jdw_v	0.9887	GATM	Glycine amidinotransferase, mitochondrial	GATM_HUMAN
2bq7_v	0.9887	F10	Coagulation factor X	FA10_HUMAN
1f86_v	0.9883	TTR	Transthyretin	TTHY_HUMAN
1jvp_v	0.988	CDK2	Cell division protein kinase 2	P24941
1hpk_v	0.9848	PLG	Plasminogen	P00747
1w8m_v	0.9845	PPIA	Peptidyl-prolyl cis-trans isomerase A	P62937
3eko_v	0.9807	HSP90AA1	Heat shock protein HSP 90-alpha	P07900
1bm6_v	0.9786	MMP3	Stromelysin-1	MMP3_HUMAN
1b09_v	0.9743	CRP	C-reactive protein	CRP_HUMAN

**FIGURE 4:**
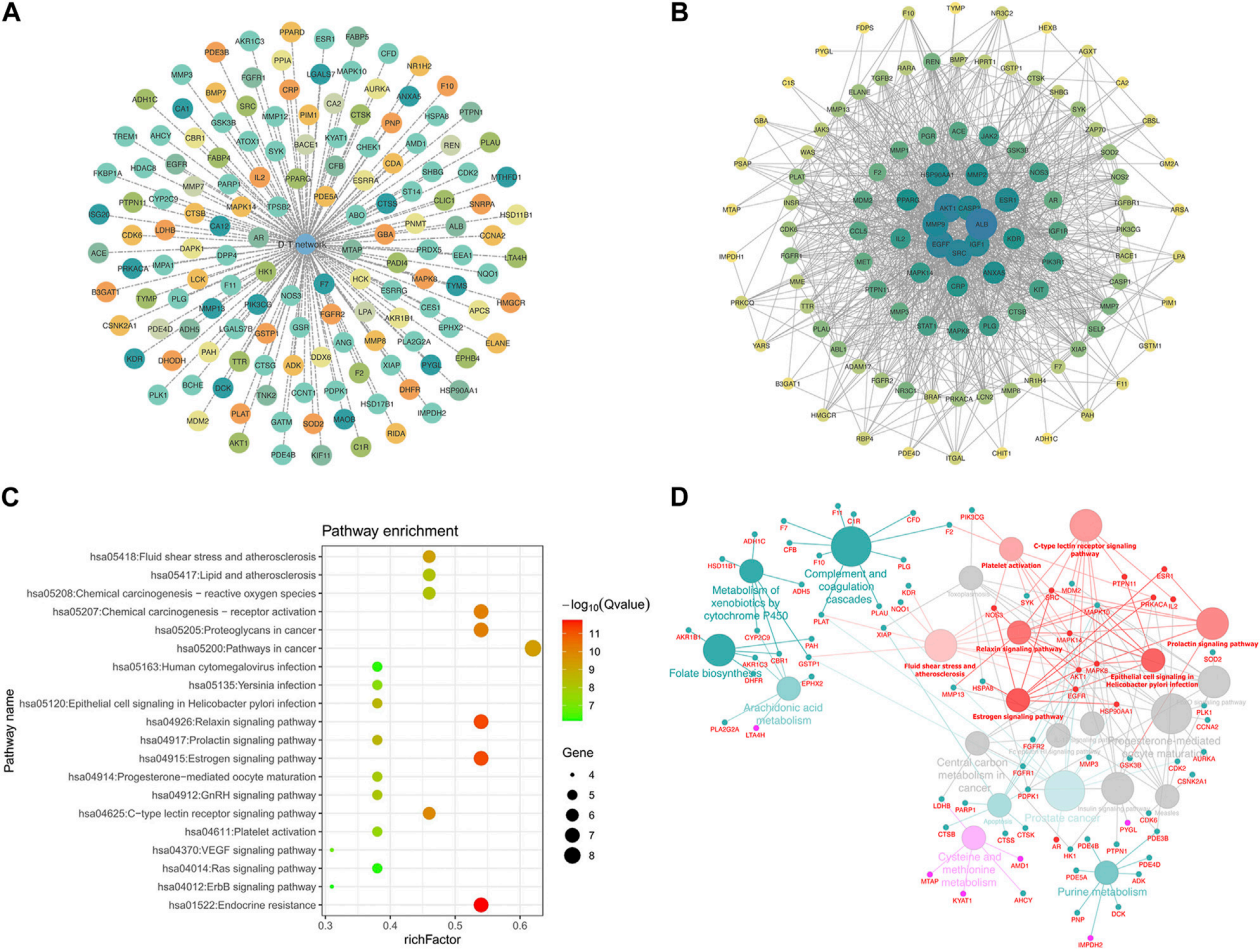
Network pharmacology targeting and pathway enrichment. **(A)** T-T network of common targets. **(B)** PPI interaction network of common targets. **(C)** KEGG pathway enrichment analysis. **(D)** Enrichment results of Hub gene pathways analyzed by ClueGO. Red indicates the enrichment results of the common targets.

A total of 147 potential targets of LPK in genes associated with bone promotion were selected to construct a target-target (T-T) network (Figure 4A). To further investigate the interactions between these targets, a protein-protein interaction (PPI) network was constructed using the potential targets as nodes (Figure 4B). The KEGG pathway enrichment analysis was performed on 13 hub genes, including MAPK8, ESR1, EGFR, MAPK14, and others, using three plugins of Cytoscape (Figure 4C). The ClueGO tool was used to further explore the correlation between KEGG pathways and the hub genes (Figure 4D). Based on this analysis, it was predicted that LPK might impact bone promotion through KEGG pathways such as Endocrine resistance, Relaxin signaling pathway, and Estrogen signaling pathway.

### 3.5 The molecular docking between LPK and core targets

**FIGURE 5:**
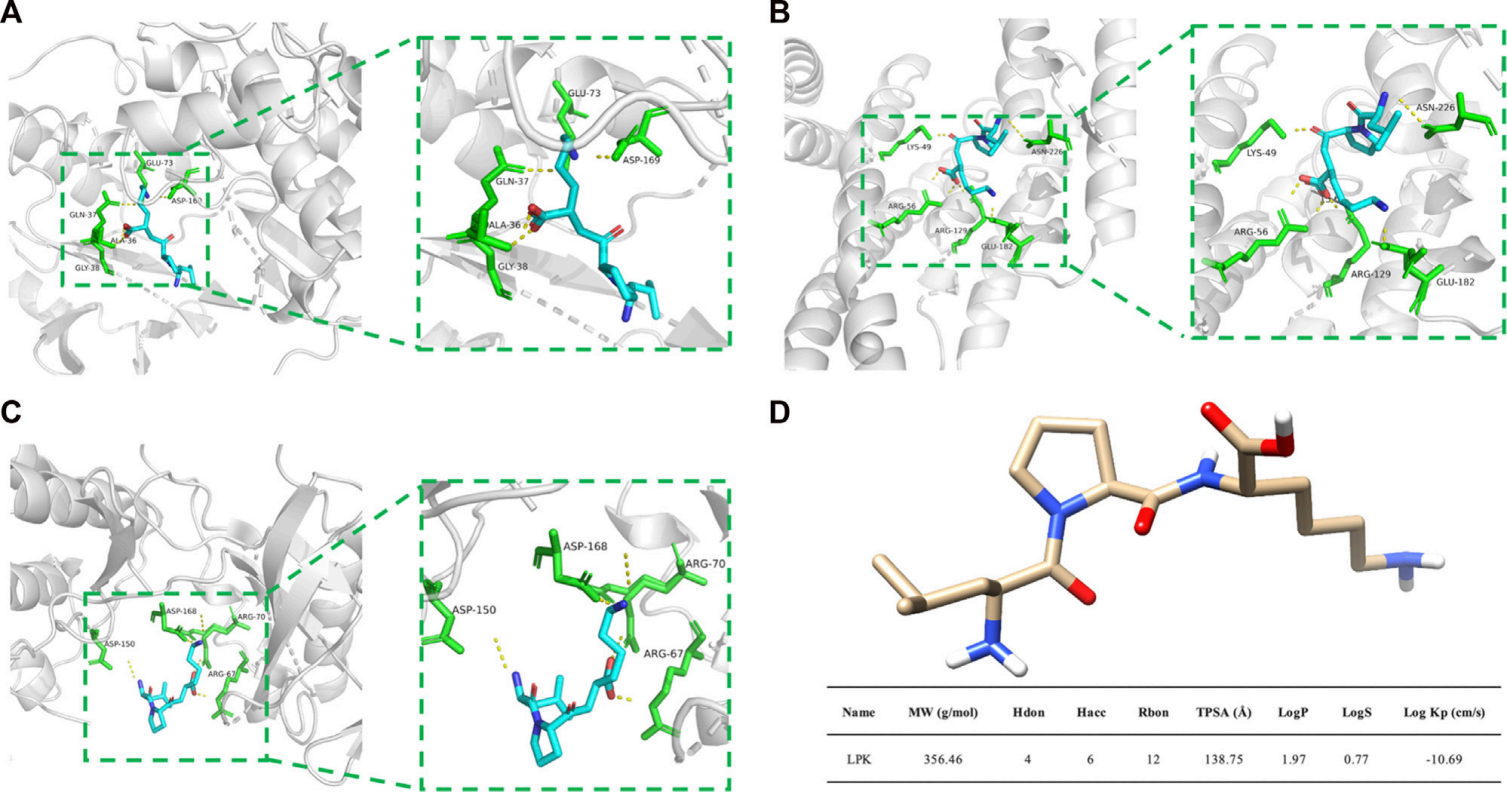
Molecular docking results and LPK structure. **(A)** The binding model of LPK and MAPK8. **(B)** The binding model of LPK and ESR1. **(C)** The binding model of LPK and MAPK14. **(D)** LPK structure and ADME results. Note: The yellow dotted lines represent the hydrogen bonds, the green rod structures represent the amino acids bound to LPK, and the blue rod structures represent the LPK structure.

The results of molecular docking are presented in Figure 5. Molecular docking is a technique used to predict the binding affinity between compounds and targets by calculating the binding free energy. A binding energy less than zero suggests that the compound and target may bind spontaneously and function, with higher negative values indicating a higher likelihood of this occurring. A more negative binding energy value also implies that the complex is more stable, and the ligand is less likely to dissociate.

Based on the analysis of the Hub genes, three representative targets were chosen for molecular docking: MAPK14, MAPK8, and ESR1. The results are displayed in Figure 5. The figure shows that each target forms a stable complex with the LPK active site, and all their binding free energies were below zero. LPK forms five hydrogen bonding interactions with GLU-73, ASP-169, GLN-37, ALA-36, and GLY-38 when bound to MAPK8 (PDB ID: 2XRW) (Figure 5A). LPK forms five hydrogen bonding interactions with LYS-49, ASN-226, ARG-56, ARG-129, and GLU-182 when bound to ESR1 (PDB ID: 7B9R) (Figure 5B). Interestingly, while both complexes form the same number of hydrogen bonds (five), the binding energy of LPK with MAPK8 (−10.511 (kcal/mol)) is much lower than that of LPK with ESR1 (−3.546 (kcal/mol)). This indicates that LPK fits better into the core of the MAPK8 active pocket, bringing a better binding pattern. LPK forms four hydrogen bonds with ASP-150, ASP-168, ARG-67, and ARG-70 when bound to MAPK14 (PDB ID: 2FST) (Figure 5C). The binding energy of LPK with MAPK14 was −8.67 kcal/mol. The results of the binding energy of LPK with the three representative targets are shown in Table 2.

**TABLE 2:** Results of molecular docking of LPK to potential targets of network pharmacology.

Target	Binding energy (kcal/mol)
MAPK8 (PDB ID: 2XRW)	−10.511
ESR1 (PDB ID: 7B9R)	−3.546
MAPK14 (PDB ID: 2FST)	−8.67

### 3.6 LPK promotes the differentiation of osteoblasts at different period

**FIGURE 6:**
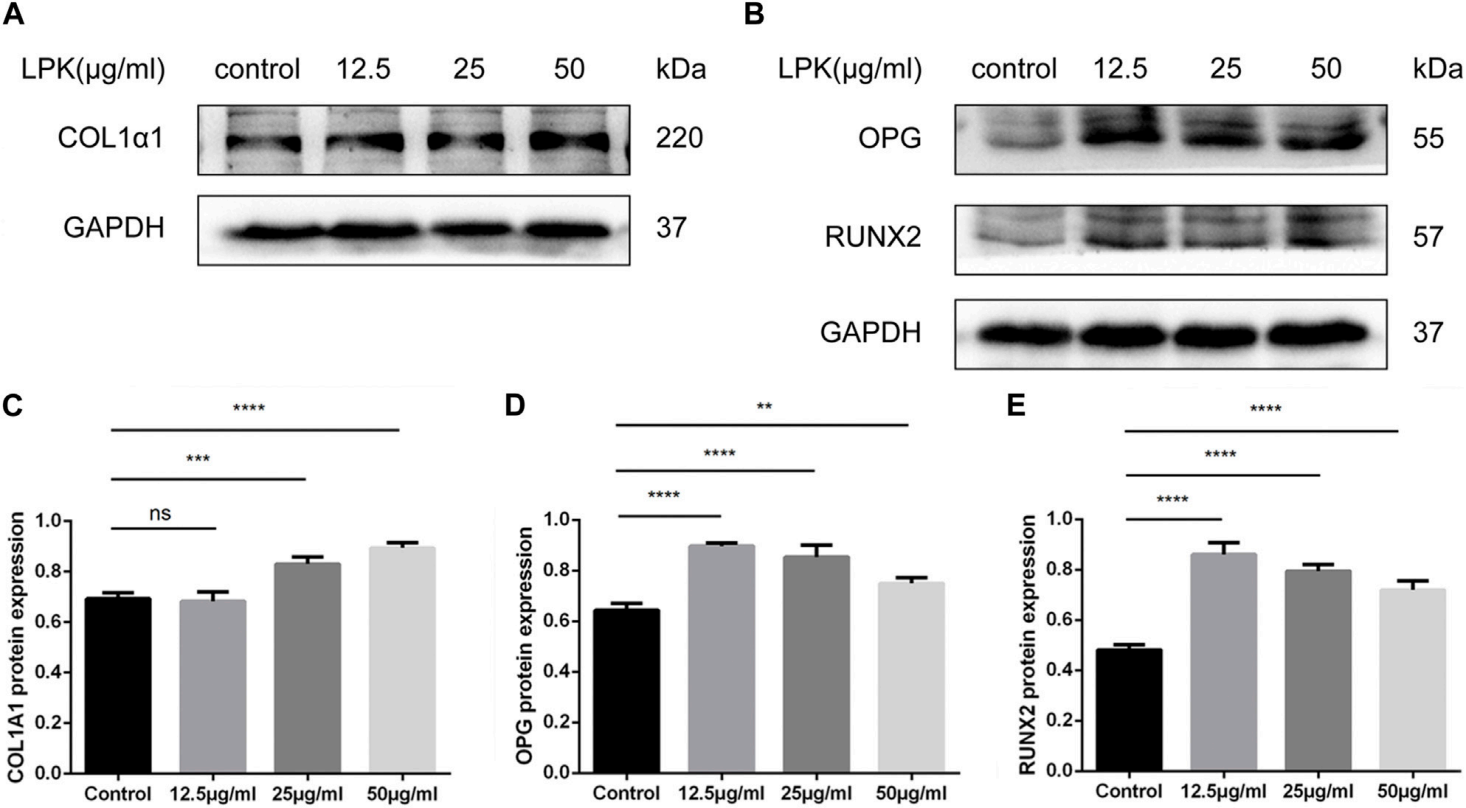
Western blot results of osteogenic markers of LPK-treated C3H10 cells. **(A)** Expression levels of type I collagen (COL1A1) in C3H10 cells. **(B)** Expression levels of osteoprotegerin (OPG) and osteogenic specific transcription factor (RUNX2) in C3H10 cells. **(C–E)** Comparative analysis of grey values for relevant protein bands, data are shown as mean ± SD (*n* = 3). Compared to the control group, differences were considered significant at *p* < 0.05 (*); *p* < 0.005 (**); *p* < 0.0005 (***); *p* < 0.00005 (****); ns: the difference was not statistically significant.

To investigate the effect of LPK on the protein expression levels of phenotypic markers of osteoblast differentiation, western blot assays were performed. As shown in Figure 6, LPK was found to promote the expression of type I collagen, RUNX2, and OPG (Figures 6A, B). The expression of type I collagen in osteoblasts was found to be dose-dependent in all LPK-treated groups (Figure 6C). Although the expression of RUNX2 and OPG slightly declined in the 25 μg/mL and 50 μg/mL LPK-treated groups, their enhancement compared to the control group was still statistically significant (Figures 6D, E). These results suggest that treatment with LPK increases the differentiation of osteoblasts.

### 3.7 LPK promotes bone development by increasing the expression of related proteins in the estrogen/MAPK signaling pathway

Based on the results of network pharmacology prediction and KEGG pathway enrichment, the potential mechanism of LPK in promoting osteoblast differentiation through the estrogen/MAPK signaling pathway was further investigated using western blotting experiments.

**FIGURE 7:**
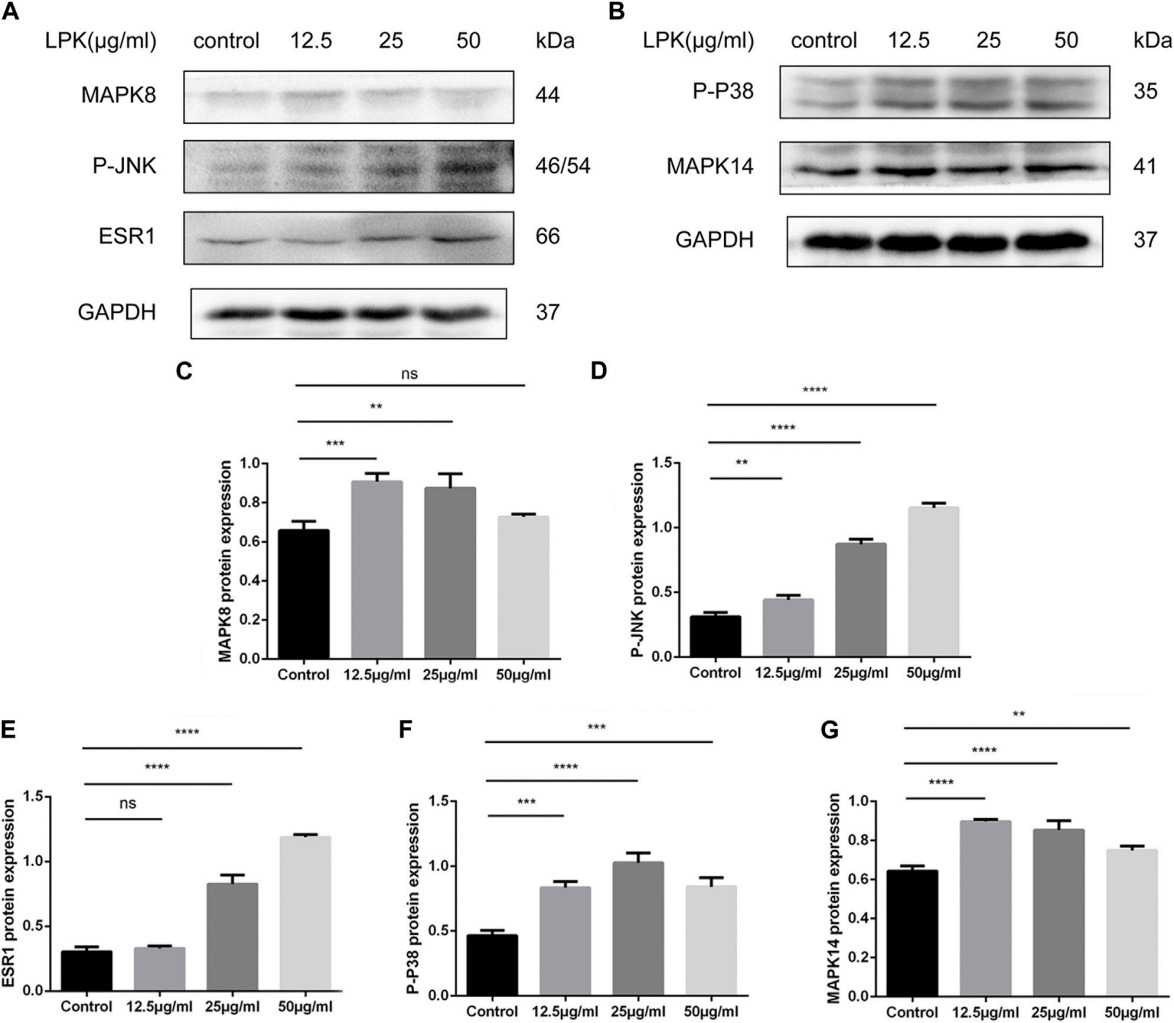
Western blot results of LPK-related pathway proteins in osteogenic-induced C3H10 cells. **(A, B)** The expression levels of estrogen receptor α (ESR1), mitogen-activated protein kinase family members (MAPK8, MAPK14), and their phosphorylated forms (P-P38, P-JNK). **(C–G)** Comparative analysis of grey values for relevant protein bands, data are shown as mean ± SD (*n* = 3). Compared to the control group, differences were considered significant at *p* < 0.05 (*); *p* < 0.005 (**); *p* < 0.0005 (***); *p* < 0.00005 (****); ns: the difference was not statistically significant.

The results of the western blotting experiments are shown in Figure 7. LPK was found to significantly increase the expression of ESR1, MAPK members, and their phosphorylated proteins, including MAPK8 (JNK1) and MAPK14 (p38 MAPK). The expression of ESR1 and P-JNK increased in a dose-dependent manner (Figures 7A, D, E). The presentation of the p-p38 protein decreased slightly in the 50 μg/mL LPK-treated group, as shown in Figure 7F. The expression levels of MAPK8 and MAPK14 decreased with increasing concentration of LPK, but their enhancement compared to the control group was still statistically significant (Figures 7A–C, G). These results suggest that LPK promotes osteoblast differentiation by differentially upregulating the expression of ESR1, MAPK8, MAPK14, and their phosphorylated proteins.

## 4 Discussion

Osteoblastic bone formation is a key aspect of bone remodeling and is a critical target for treating osteoporosis. Many studies have shown that natural antioxidant compounds such as flavonoids (Xue et al., 2021), polyphenols (Welch and Hardcastle, 2014; Jiang et al., 2020), and small molecular weight peptides (Heo et al., 2018; Shi et al., 2020) can stimulate osteoblast differentiation, promote bone formation, and thus play an anti-osteoporosis effect. Therefore, finding candidate drugs with bone formation effects from antioxidant compounds is a current area of focus in anti-osteoporosis research.

Previously, our study (Liu et al., 2022) showed that LPK can inhibit H2O2-induced cell death by blocking ROS activation. Additionally, ADME calculations revealed that LPK has favorable drug-like properties. However, the effect of LPK on bone formation was not fully understood. The current study’s results demonstrate that LPK effectively stimulates differentiation and mineralization in pre-osteoblast C3H10 cells and zebrafish larvae. These findings suggest that LPK is a potential candidate for treating osteoporosis.

Osteoblasts are bone-forming cells that differentiate from multipotent mesenchymal stem cells (MSCs) (Huang et al., 2007) under the influence of key osteogenic transcription factors such as distal-less homeobox 5 (Dlx5), runt-related transcription factor 2 (Runx2) (Komori, 2019) and osteogenic factors. They primarily participate in matrix production and mineralization to form strong bones. These transcription factors independently or in combination stimulate the expression of osteogenic genes such as alkaline phosphatase (ALP), type I collagen, and osteocalcin, to promote bone mineralization. This research showed that different concentrations of LPK-stimulated osteoblasts, and significantly promoted osteogenic differentiation and mineralization. Furthermore, calcein and alizarin red double staining of zebrafish larvae showed that LPK notably contributes to the formation of new bones in zebrafish larvae. This bone mineralization effect was enhanced with an increase in LPK concentration. Immunofluorescence experiments confirmed that COL1A1 actively participated in bone mineralization in zebrafish. ZNS5 is a cell surface antigen used as an osteoblast-specific marker, although ZNS5 did not show a corresponding trend after administration, the expression levels were above the control group. This indicates that LPK is positively correlated with osteoblast differentiation and mineralization. The results suggest that up-regulating the expression of COL1A1 in osteoblasts is a crucial factor in LPK-promoting bone formation.

In this study, both *in vivo* and *in vitro* bone formation experiments have shown that LPK can promote bone remodeling by stimulating bone formation, but the underlying mechanism is not yet known. Therefore, network pharmacology methods were used to predict the core targets and signaling pathways of LPK that actively act on osteoblasts. Three Cytoscape plug-ins, CentiScaPe, Mcode, and cytoHubba, were used to accurately screen the range and the intersection of the results of the three methods as the core target of LPK promoting bone development. 13 Hub genes, including MAPK14, MAPK8, and ESR1, were collected and were highly correlated with the MAPK signaling pathway and the estrogen signaling pathway. Through molecular docking, our results further verified that LPK could closely interact with MAPK8, MAPK14, and ESR1 in regulating osteoblast differentiation through the estrogen/MAPK signaling pathway.

The classic MAPKs signal pathway (Ge et al., 2007), which includes ERK1/2 signaling, JNK1/2/3 signaling, and p38 MAPK cascade signaling, has been shown to play a crucial role in skeletal development and bone remodeling, particularly in regard to osteoblast differentiation and mineralization. In this study, MAPK8 and MAPK14 were identified as the key targets of LPK acting on osteoblasts in the MAPK pathway. The results of protein expression levels showed that an increase in the phosphorylated forms of MAPK8 and MAPK14 correlated with an increase in gene expression levels necessary for mineralization (such as RUNX2, COL1A1, and OPG). This indicates that the mitogen-activated protein kinase MAPK signaling pathway is closely related to protein phosphorylation on osteoblast factors and their pathways. ALP and COL1A1 are markers of early osteoblast differentiation. They are involved in the deposition of phosphate and in promoting the maturation of the extracellular matrix in the early stage of osteoblasts formation. OPG is a non-collagenous protein secreted during osteoblast differentiation that can specifically combine with calcium and phosphate to form hydroxyapatite crystals (Ma et al., 2017), which is a marker of late osteoblast differentiation. As these markers increase with the phosphorylation levels of MAPK8 and MAPK14, it suggests that the MAPK signaling pathway may be involved in LPK-mediated early or late osteoblast differentiation.

ESR1, also known as ERα, encodes one of the two subtypes of the estrogen receptor. It is widely expressed in bones and plays a role in bone metabolism (Kousteni et al., 2001). ESR1 binds to estrogen via the G protein-coupled estrogen receptor 1 (GPER1), activating the ERK/MAPK signaling pathway and promoting osteoblast differentiation (Lara-Castillo, 2021). The activation of the MAPK signaling pathway can also activate ERα by phosphorylation so that the cascade effect further amplifies its signaling molecules. This signaling pathway also leads to the phosphorylation and activation of ERα, amplifying its signaling molecules.

**FIGURE 8:**
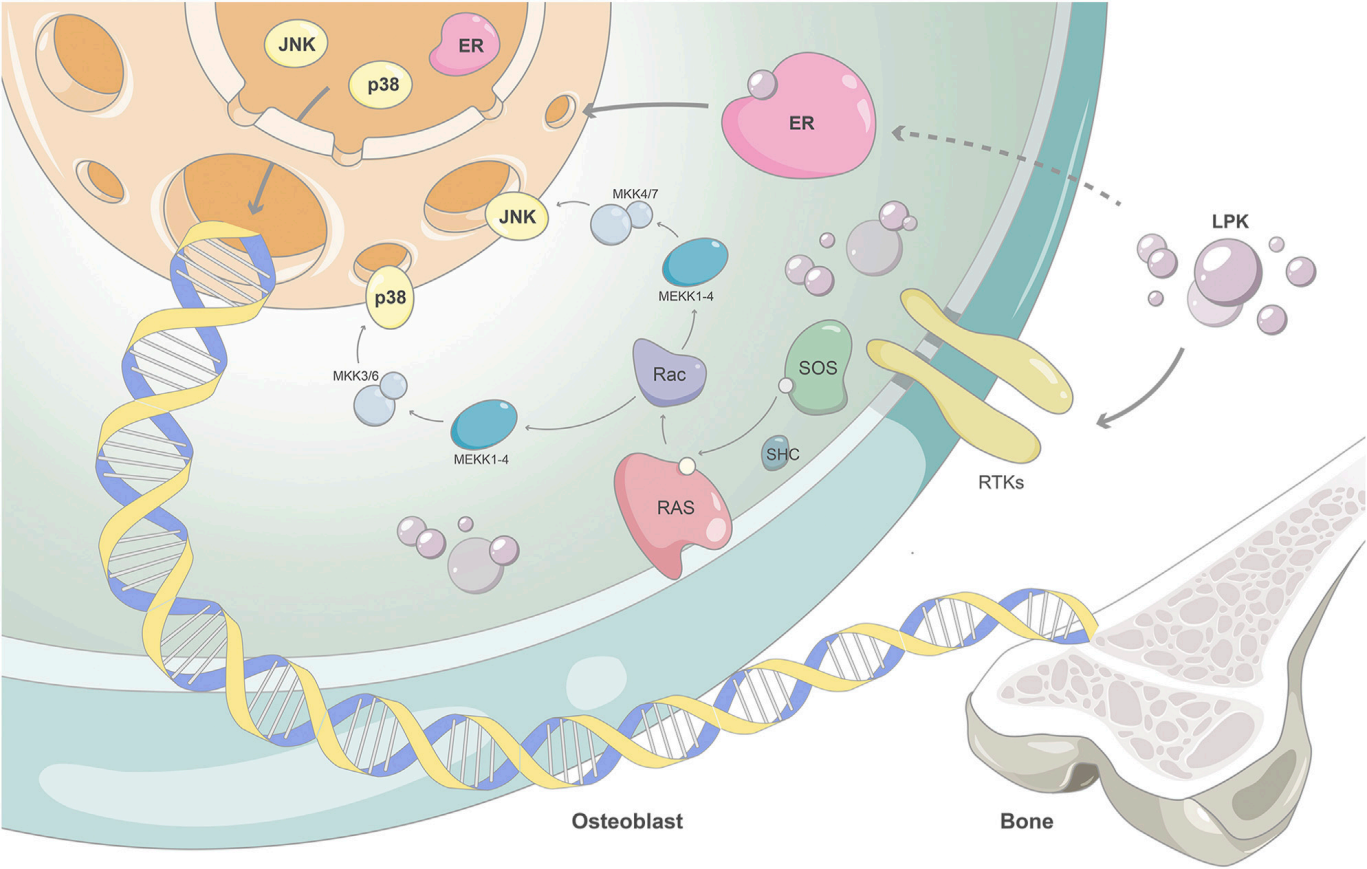
The proposed mechanism of action of Sipunculus nudus tripeptide LPK in promoting bone formation.

Our results showed that the expression of ESR1 increased in a dose-dependent manner with the intervention of LPK. Additionally, the expression levels of phosphorylated proteins of MAPK8 and MAPK14 also increased. This suggests that LPK can stimulate the activation of the estrogen/MAPK signaling axis by increasing the expression of ESR1 protein, this in turn participates in the activation of the MAPK signaling pathway and promotes osteoblast differentiation. This is illustrated in Figure 8 as the proposed mechanism of action of Sipunculus nudus tripeptide LPK in promoting bone formation.

## 5 Conclusion

In summary, our study indicates that Sipunculus nudus peptide LPK positively affects the proliferation and differentiation of mouse pre-osteoblast cells (C3H10). Specifically, LPK accelerates the deposition of phosphate and promotes the maturation of the extracellular matrix in the early stage of osteoblasts formation. Additionally, LPK promotes osteoblastic bone formation by stimulating osteoblast differentiation and mineralization, potentially through activating the p38/JNK/MAPK pathway and its downstream effectors such as COL1A1, RUNX2, and ALP. Furthermore, our data suggest that LPK may also have a beneficial effect on osteoblast formation via the estrogen signaling pathway. These findings suggest that LPK has therapeutic potential for the treatment of osteoporosis and provides a promising candidate for further research in this area.

## Data Availability

The datasets presented in this study can be found in online repositories. The names of the repository/repositories and accession number(s) can be found in the article.

## References

[B1] Bader G. D. Hogue C. W. V. (2003). An automated method for finding molecular complexes in large protein interaction networks. BMC Bioinforma. 4 (1), 2. 10.1186/1471-2105-4-2 PMC149346 12525261

[B2] Bennett M. L. Bennett F. C. Liddelow S. A. Ajami B. Zamanian J. L. Fernhoff N. B. (2016). New tools for studying microglia in the mouse and human CNS. Proc. Natl. Acad. Sci. 113 (12), E1738–E1746. 10.1073/pnas.1525528113 26884166 PMC4812770

[B3] Berman H. M. Westbrook J. Feng Z. Gilliland G. Bhat T. N. Weissig H. (2000). The protein data bank. Nucleic Acids Res. 28 (1), 235–242. 10.1093/nar/28.1.235 10592235 PMC102472

[B4] Bindea G. Mlecnik B. Hackl H. Charoentong P. Tosolini M. Kirilovsky A. (2009). ClueGO: A Cytoscape plug-in to decipher functionally grouped gene ontology and pathway annotation networks. Bioinforma. Oxf. Engl. 25 (8), 1091–1093. 10.1093/bioinformatics/btp101 PMC2666812 19237447

[B5] Bonte M.-A. El Idrissi F. Gressier B. Devos D. Belarbi K. (2021). Protein network exploration prioritizes targets for modulating neuroinflammation in Parkinson’s disease. Int. Immunopharmacol. 95, 107526. 10.1016/j.intimp.2021.107526 33756233

[B6] Chin C.-H. Chen S.-H. Wu H.-H. Ho C.-W. Ko M.-T. Lin C.-Y. (2014). cytoHubba: identifying hub objects and sub-networks from complex interactome. BMC Syst. Biol. 8 (4), S11. 10.1186/1752-0509-8-S4-S11 25521941 PMC4290687

[B7] Consortium U. (2019). UniProt: A worldwide hub of protein knowledge. Nucleic Acids Res. 47 (1), D506–D515. 10.1093/nar/gky1049 30395287 PMC6323992

[B8] Diskin R. Lebendiker M. Engelberg D. Livnah O. (2007). Structures of p38alpha active mutants reveal conformational changes in L16 loop that induce autophosphorylation and activation. J. Mol. Biol. 365 (1), 66–76. 10.1016/j.jmb.2006.08.043 17059827

[B9] Dong L. F. Zhang Q. Tong T. Xu M. Z. Chen J. H. (2012). Amino acid composition of peanut worm Sipunculus nudus at different growth stages. South China Fish. Sci. 8 (5), 60–65. 10.3969/j.issn.2095-0780.2012.05.009

[B10] Friesner R. A. Murphy R. B. Repasky M. P. Frye L. L. Greenwood J. R. Halgren T. A. (2006). Extra precision Glide: Docking and scoring incorporating a model of hydrophobic enclosure for Protein−Ligand complexes. J. Med. Chem. 49 (21), 6177–6196. 10.1021/jm051256o 17034125

[B11] Garai Á. Zeke A. Gógl G. Törő I. Fördős F. Blankenburg H. (2012). Specificity of linear motifs that bind to a common mitogen-activated protein kinase docking groove. Sci. Signal. 5 (245), ra74. 10.1126/scisignal.2003004 23047924 PMC3500698

[B12] Ge C. X. Xiao G. Z. Jiang D. Franceschi R. T. (2007). Critical role of the extracellular signal–regulated kinase–MAPK pathway in osteoblast differentiation and skeletal development. J. Cell Biol. 176 (5), 709–718. 10.1083/jcb.200610046 17325210 PMC2064027

[B13] Heo S.-Y. Ko S.-C. Nam S. Y. Oh J. Kim Y.-M. Kim J.-I. (2018). Fish bone peptide promotes osteogenic differentiation of MC3T3-E1 pre-osteoblasts through upregulation of MAPKs and Smad pathways activated BMP-2 receptor. Cell Biochem. Funct. 36 (3), 137–146. 10.1002/cbf.3325 29392739

[B14] Huang W. Yang S. Y. Shao J. Z. Li Y. P. (2007). Signaling and transcriptional regulation in osteoblast commitment and differentiation. Front. Biosci. a J. Virtual Libr. 12, 3068–3092. 10.2741/2296 PMC3571113 17485283

[B15] Jeon H. H. Yang C.-Y. Shin M. K. Wang J. Y. Patel J. H. Chung C.-H. (2021). Osteoblast lineage cells and periodontal ligament fibroblasts regulate orthodontic tooth movement that is dependent on Nuclear Factor-kappa B (NF-kB) activation. Angle Orthod. 91 (5), 664–671. 10.2319/031520-182.1 33852725 PMC8376154

[B16] Jiang Y. X. Luo W. Q. Wang B. Wang X. Y. Gong P. Xiong Y. (2020). Resveratrol promotes osteogenesis via activating SIRT1/FoxO1 pathway in osteoporosis mice. Life Sci. 246, 117422. 10.1016/j.lfs.2020.117422 32057903

[B17] Kanehisa M. Furumichi M. Sato Y. Kawashima M. Ishiguro-Watanabe M. (2022). KEGG for taxonomy-based analysis of pathways and genomes. Nucleic Acids Res. 51, D587–D592. 10.1093/nar/gkac963 PMC9825424 36300620

[B18] Komori T. (2019). Regulation of proliferation, differentiation and functions of osteoblasts by Runx2. Int. J. Mol. Sci. 20 (7), 1694. 10.3390/ijms20071694 30987410 PMC6480215

[B19] Kousteni S. Bellido T. Plotkin L. I. O'Brien C. A. Bodenner D. L. Han L. (2001). Nongenotropic, sex-nonspecific signaling through the estrogen or androgen receptors: Dissociation from transcriptional activity. Cell 104 (5), 719–730. 10.1016/S0092-8674(01)00268-9 11257226

[B20] Lara-Castillo N. (2021). Estrogen signaling in bone. Appl. Sci. 11 (10), 4439. 10.3390/app11104439 PMC11526471 39483629

[B21] Li L. L. Wei Q. Y. Wang Y. F. He Z. M. Gao Y. G. Ma J. S. (2017). Research progress of FGF/FGFR signaling regulating osteoblast differentiation. China Biotechnol. 37 (6), 107–113. 10.13523/j.cb.20170616

[B22] Lipinski C. A. (2004). Lead- and drug-like compounds: The rule-of-five revolution. Drug Discov. Today Technol. 1 (4), 337–341. 10.1016/j.ddtec.2004.11.007 24981612

[B23] Liu H. Cai X. Huang M. Wang T. Li L. Luo H. (2022). Dual bioactivity of angiotensin converting enzyme inhibition and antioxidant novel tripeptides from *Sipunculus nudus* L. and their related mechanism analysis for antihypertention. Int. J. Pept. Res. Ther. 29 (1), 3. 10.1007/s10989-022-10470-6

[B24] Liu Z. Tang X. N. Wu K. F. Lin Q. X. Liu H. Li Q. (2021). Isolation and identification of oligopeptides from squares siphon worm and its effect on promoting bone growth. Sci. Technol. Food Industry 42 (20), 334. 10.13386/j.issn1002-0306.2020120263

[B25] Ma X. N. Ma C. X. Shi W. G. Zhou J. Ma H. P. Gao Y. H. (2017). Primary cilium is required for the stimulating effect of icaritin on osteogenic differentiation and mineralization of osteoblasts *in vitro* . J. Endocrinol. Investigation 40 (4), 357–366. 10.1007/s40618-016-0568-8 27770387

[B26] Madhavi Sastry G. Adzhigirey M. Day T. Annabhimoju R. Sherman W. (2013). Protein and ligand preparation: Parameters, protocols, and influence on virtual screening enrichments. J. Computer-Aided Mol. Des. 27 (3), 221–234. 10.1007/s10822-013-9644-8 23579614

[B27] Murshed M. (2018). Mechanism of bone mineralization. Cold Spring Harb. Perspect. Med. 8 (12), a031229. 10.1101/cshperspect.a031229 29610149 PMC6280711

[B28] O'Boyle N. M. Banck M. James C. A. Morley C. Vandermeersch T. Hutchison G. R. (2011). Open Babel: An open chemical toolbox. J. Cheminformatics 3 (1), 33. 10.1186/1758-2946-3-33 PMC3198950 21982300

[B29] Pettersen E. F. Goddard T. D. Huang C. C. Couch G. S. Greenblatt D. M. Meng E. C. (2004). UCSF Chimera--a visualization system for exploratory research and analysis. J. Comput. Chem. 25 (13), 1605–1612. 10.1002/jcc.20084 15264254

[B30] Piñero J. Ramírez-Anguita J. M. Saüch-Pitarch J. Ronzano F. Centeno E. Sanz F. (2019). The DisGeNET knowledge platform for disease genomics: 2019 update. Nucleic Acids Res. 48 (D1), D845–D855. 10.1093/nar/gkz1021 PMC7145631 31680165

[B31] Scardoni G. Tosadori G. Faizan M. Spoto F. Fabbri F. Laudanna C. (2014). Biological network analysis with CentiScaPe: Centralities and experimental dataset integration. F1000Research 3, 139. 10.12688/f1000research.4477.2 26594322 PMC4647866

[B32] Shannon P. Markiel A. Ozier O. Baliga N. S. Wang J. T. Ramage D. (2003). Cytoscape: A software environment for integrated models of biomolecular interaction networks. Genome Res. 13 (11), 2498–2504. 10.1101/gr.1239303 14597658 PMC403769

[B33] Shi P. J. Fan F. J. Chen H. Xu Z. Cheng S. Z. Lu W. H. (2020). A bovine lactoferrin-derived peptide induced osteogenesis via regulation of osteoblast proliferation and differentiation. J. Dairy Sci. 103 (5), 3950–3960. 10.3168/jds.2019-17425 32197844

[B34] Sijbesma E. Hallenbeck K. K. Andrei S. A. Rust R. R. Adriaans J. M. C. Brunsveld L. (2021). Exploration of a 14-3-3 PPI pocket by covalent fragments as stabilizers. ACS Med. Chem. Lett. 12 (6), 976–982. 10.1021/acsmedchemlett.1c00088 34136078 PMC8201753

[B35] Stelzer G. Dalah I. Stein T. I. Satanower Y. Rosen N. Nativ N. (2011). *In-silico* human genomics with GeneCards. Hum. Genomics 5 (6), 709–717. 10.1186/1479-7364-5-6-709 22155609 PMC3525253

[B36] Szklarczyk D. Gable A. L. Nastou K. C. Lyon D. Kirsch R. Pyysalo S. (2021). The STRING database in 2021: Customizable protein-protein networks, and functional characterization of user-uploaded gene/measurement sets. Nucleic Acids Res. 49 (1), D605–D612. 10.1093/nar/gkaa1074 33237311 PMC7779004

[B37] Wang G. F. Dong J. C. Duan X. H. (2012). Concept of ‘kidney’ in TCM and mechanism, method, prescription, drug & application of kidney-invigorating. China J. Traditional Chin. Med. Pharm. 27 (12), 3112–3115.

[B38] Wang X. Shen Y. H. Wang S. W. Li S. L. Zhang W. L. Liu X. F. (2017). PharmMapper 2017 update: A web server for potential drug target identification with a comprehensive target pharmacophore database. Nucleic Acids Res. 45 (1), W356–W360. 10.1093/nar/gkx374 28472422 PMC5793840

[B39] Welch A. A. Hardcastle A. C. (2014). The effects of flavonoids on bone. Curr. Osteoporos. Rep. 12 (2), 205–210. 10.1007/s11914-014-0212-5 24671371

[B40] Wickham H. (2016). “Data analysis,” in ggplot2 (Germany: Springer), 189–201.

[B41] Wilson L. Matsudaira P. (1998). “The zebrafish, biology,” in Methods in cell biology William Detrich I. H. Westerfield M. Zon L. I. (Cambridge, Massachusetts: Academic Press), 308.

[B42] Wu M. R. Deng L. F. Zhu G. C. Li Y. P. (2010). G Protein and its signaling pathway in bone development and disease. Front. Biosci. (Landmark Ed. 15 (3), 957–985. 10.2741/3656 20515736

[B43] Xue F. Zhao Z. L. Gu Y. P. Han J. X. Ye K. Q. Zhang Y. (2021). 7,8-Dihydroxyflavone modulates bone formation and resorption and ameliorates ovariectomy-induced osteoporosis. ELife 10, e64872. 10.7554/eLife.64872 34227467 PMC8285109

[B44] Zhang C. X. Dai Z. R. Cai Q. X. (2011). Anti-inflammatory and anti-nociceptive activities of Sipunculus nudus L. extract. J. Ethnopharmacol. 137 (3), 1177–1182. 10.1016/j.jep.2011.07.039 21807085

[B45] Zhang Q. (2003). Research on the preparation of bioactive peptides. J. Animal Sci. Veterinary Med. 22 (3), 20–21.

[B46] Zheng M. (2010). Study of multifunctional plant small molecule peptides. dissertation/master's thesis. China: Nanchang University.

[B47] Zhou Y. Y. Zhou B. Pache L. Chang M. Khodabakhshi A. H. Tanaseichuk O. (2019). Metascape provides a biologist-oriented resource for the analysis of systems-level datasets. Nat. Commun. 10 (1), 1523. 10.1038/s41467-019-09234-6 30944313 PMC6447622

